# Comparison of Nalbuphine Versus Clonidine as an Adjuvant to Intrathecal Hyperbaric Bupivacaine in Orthopedic Lower Limb Surgeries: A Randomized Controlled Double-Blind Study

**DOI:** 10.7759/cureus.42857

**Published:** 2023-08-02

**Authors:** Heena Agrawal, Sujata Chaudhary, Rashmi Salhotra

**Affiliations:** 1 Department of Anesthesia, Late Bisahu Das Mahant Memorial Medical College, Korba, Jhagarha, IND; 2 Department of Anesthesia, Vardhman Mahavir Medical College and Safdarjung Hospital, New Delhi, IND; 3 Department of Anesthesia, University College of Medical Sciences, New Delhi, IND

**Keywords:** trauma, orthopaedic, spinal anaesthesia, hyperbaric bupivacaine, intrathecal, clonidine, nalbuphine

## Abstract

Background: Nalbuphine and clonidine are used as adjuvants to intrathecal local anesthetics, but studies on their comparative efficacy have shown inconsistent results. This study aimed to compare nalbuphine, clonidine, or normal saline as adjuvants to intrathecal hyperbaric bupivacaine in orthopedic lower limb surgeries.

Method: Sixty-three American Society of Anesthesiologists (ASA) I/II patients between 18 and 60 years old with lower limb fractures were randomized into three groups (n=21 each). Patients with contraindications to spinal block, bilateral lower limb fractures, or long-term opioid therapy were excluded. The subarachnoid block was given in L3-L4 interspace. Group N received 1 mg of nalbuphine, group Cl received 30 mcg of clonidine, and group C received 0.5 ml of normal saline with 15 mg (0.5%) of hyperbaric bupivacaine. Sensory and motor block characteristics, hemodynamic variables and side effects were noted, and the data were analyzed using Student’s t-test, Mann-Whitney test, Chi-square test, and ANOVA followed by Tukey’s test.

Results: Patients receiving intrathecal nalbuphine (group N) and clonidine (group Cl) had a faster onset of the sensory and motor block than controls (group C) (p=0.000). The time to two-segment regression was more prolonged in group Cl when compared to group N (p=0.000). Duration of spinal analgesia was 216.75 ± 25.96 minutes, 292.86 ± 24.92 minutes, and 178.50 ± 16.06 minutes in groups N, Cl, and C, respectively (p=0.000). The 24-hour rescue analgesic requirement was maximum in group C and least in group Cl (p=0.000). The three groups were comparable to each other in terms of side effects.

Conclusion: Clonidine was found to be superior to nalbuphine as an intrathecal adjuvant with no significant side effects.

## Introduction

Lower limb orthopedic surgeries continue to remain an enigma because of their requirement for prolonged analgesia along with the need for early ambulation. Therefore, the subarachnoid blockade is the preferred mode of anesthesia as it has a rapid onset of action, provides effective pain relief and is also cost-effective and provides a better safety profile in terms of avoiding side effects of multiple drugs [1-3]. Local anesthetic drugs have been commonly used via the intrathecal route, but due to the limited duration of action of bupivacaine, patients experience early post-operative pain. Various adjuvants are being used with local anesthetics to prolong the duration of analgesia and minimize the adverse effects of high doses of local anesthetics [4-9].

Nalbuphine is a highly lipid-soluble synthetic opioid. It is an agonist at κ receptors and an antagonist at µ receptors. It has been used intrathecally in variable doses with variable durations of analgesia. Higher doses were associated with a greater incidence of side effects like nausea, vomiting, and pruritis. The ideal intrathecal dose for providing adequate analgesia without significant side effects is still unknown [10-15].

Clonidine is an alpha1 and alpha2 adrenoreceptor agonist with predominant alpha2 action. It produces analgesia by activation of descending medullospinal noradrenergic tract and central sympatholysis at the presynaptic ganglion. It is associated with various side effects like hypotension and bradycardia. It has been used intrathecally in various doses ranging from 15 mcg to 450 mcg, but no ideal dose has yet been found. It is commonly used in the dose range of 15 to 60 mcg [16-18].

On a literature search, there are limited studies comparing nalbuphine and clonidine as intrathecal adjuvants [4,16,19]. The present study was designed to compare the effects of 1 mg nalbuphine and 30 mcg clonidine as an intrathecal adjuvant to hyperbaric bupivacaine in orthopedic lower limb surgery. Our primary outcome measure was to determine the duration of spinal analgesia following intrathecal injection (defined as the time from intrathecal injection till the time to the requirement of first rescue analgesia (visual analog scale (VAS) ≥3). As our secondary objective, we observed characteristics of sensory and motor block, postoperative pain assessment, and total 24-hour analgesic requirements.

## Materials and methods

Study design and subjects

The study was conducted between October 2018 and April 2020 after approval from the Institutional Ethics Committee of the University College of Medical Sciences, University of Delhi (IEC-HR/2018/36/8R). The study was registered with www.ctri.nic.in before participant enrollment (Registration number: CTRI/2018/12/016742). After obtaining written informed consent from the participants, sixty-three American Society of Anesthesiologists (ASA) I/II patients between 18 and 60 years, with a height between 150 and 180 cm undergoing orthopedic lower limb surgeries were included in this prospective, double-blind, randomized study. Patients with contraindications to central neuraxial block, weight >120 kg, history of chronic pain or long-term opioid use, known allergy to drugs, or multiple fractures precluding assessment of anesthesia were excluded.

Pre-, intra-, and post-operative methodology

After a routine pre-anesthetic checkup, patients were informed about the procedure of spinal anesthesia and were introduced to the concept of the visual analog scale (VAS). A tablet of alprazolam of 0.5 mg was given orally the night before the surgery. On the day of the surgery, baseline heart rate (HR), systolic blood pressure (SBP), diastolic blood pressure (DBP), mean blood pressure (MBP), peripheral oxygen saturation (SpO_2_), and electrocardiogram (ECG) were recorded. After securing a wide-bore 18G intravenous cannula, co-loading was done with 15 ml/kg of Ringer’s lactate solution.

Under all aseptic precautions, the subarachnoid block was performed via the midline approach at L3-4/L4-5 interspace using a 25G Quincke’s (MediSpine spinal needle, Global Medikit Limited, India) needle with the patient in a sitting position. Patients were randomized to one of the three groups based on a computer-generated table of random numbers. Allocation concealment was done using sequentially numbered sealed opaque envelopes. Group N received 0.5% hyperbaric bupivacaine (15 mg) with 0.5 ml of nalbuphine (1 mg). Group Cl received 0.5% hyperbaric bupivacaine (15 mg) with 0.5 ml clonidine (30 mcg), and group C received 0.5% hyperbaric bupivacaine (15 mg) with 0.5 ml normal saline. The intrathecal drug was prepared by an anesthesiologist not involved in the study. Immediately, patients were placed supine, and oxygen was administered at 5 L/minutes by face mask. Ringer lactate was used as a maintenance and replacement fluid. Blood loss until the maximum allowable limit was replaced by crystalloids in the ratio of 3:1. Mephentermine 6 mg was given to treat hypotension (SBP <90 mmHg or >20% fall from baseline). Atropine was given to treat bradycardia (HR <50 bpm or >20% fall from baseline).

The time of intrathecal injection was noted, and all observations were made using this time as '0’ minutes. The sensory block was assessed by 27G hypodermic needle every two minutes from '0’ minutes till four consecutive tests remain at the same dermatome, and then every 15 minutes till the initial two-segment regression of the block and every half an hour till regression to S1. Onset of sensory block (T10), highest level of sensory dermatome blocked and duration of sensory block (time taken for two-segment regression) were noted. The motor block was assessed by using the modified Bromage grade (MBG). The intensity of the motor block was assessed every two minutes until a complete motor block was achieved. Onset (MBG-3) and duration of the motor block (time taken for complete recovery, MBG-0) were noted. Pain scoring was done using the visual analog scale (VAS) at a 30-minute interval for the first two hours and the fourth, eighth, 12th, and 24th hours in the postoperative period. The duration of analgesia was taken as the time from intrathecal injection until the requirement of first rescue analgesia (VAS ≥3). Injection of diclofenac 75 mg was given intravenously as the first rescue analgesic, and if VAS was persistently ≥3 after half an hour of diclofenac, then injection of paracetamol 1 gm was given.

Hemodynamic parameters like HR, SBP, DBP, MBP, and SpO_2_ were monitored every five minutes for the first 15 minutes, then every 15 minutes till the end of the surgery, every 30 minutes till two hours postoperatively, and then hourly till complete recovery. Sedation scoring was done using the Ramsay sedation score every 30 minutes intraoperatively and hourly for two hours in the postoperative period. Patients were monitored for side effects like pruritis, hypotension, bradycardia, nausea, vomiting, sedation, and respiratory depression. 

Sample size

The sample size estimation was based on the duration (minutes) of analgesia among groups [17]. For the sample size calculation, we have defined a mean difference of 8.56 with a 10.01 standard deviation. We have calculated a sample size with a 95% confidence interval, 80% power, and an alpha level of 0.05.

On comparison of two mean formula, N=size per group; SD=standard deviation=10.01, mean difference=223.16−214.6=8.56, Z α/2=Z 0.05/2=Z 0.025=1.96. From Z table at type I error of 5 Z β=Z 0.20=0.84 at 80% power

= 2 (1.96 + 0.84) 2 (10.01) 2/(8.56) 2 = 15.68 × 100.2001/73.27 = 1571.13/73.27 = 21.44 = 21

Hence the sample size can be taken as 21 in each group.

Statistical analysis

Categorical variables are presented in numbers, and continuous variables are presented as mean ± SD and median. Between the three groups, the comparison was done by one-way ANOVA followed by Tukey’s test. Qualitative parameters were compared using the Chi-square test. All statistical analysis was done in SPSS version 20.0 (IBM Corp., New York, USA). A p-value of <0.05 was considered significant.

## Results

**Figure 1:**
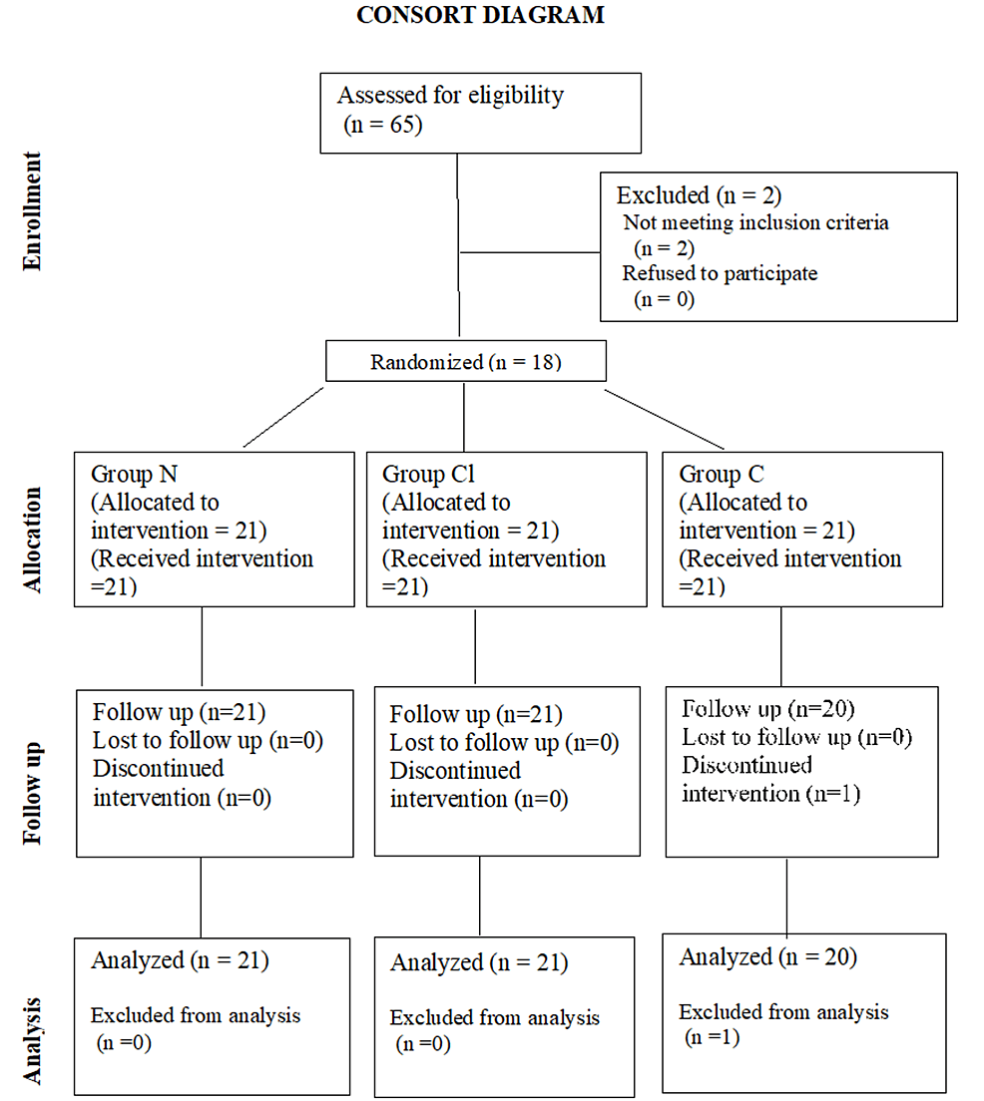
CONSORT flow diagram. CONSORT: Consolidated Standards of Reporting Trials.

Figure 1 shows the flow of patients through the trial in the Consolidated Standards of Reporting Trials (CONSORT) diagram.

The demographic characteristics of the three groups are shown in Table 1. All three groups were comparable to each other.

**Table 1:** Demographic profile in three groups.

Parameters		Group N (nalbuphine)	Group Cl (clonidine)	Group C (control)	p-value
Age (years)	Mean ± SD	35.05 ± 8.65	34.0 ± 12.23	34.40 ± 10.21	0.94
Weight (kg)	Mean ± SD	66.19 ± 8.37	68.05 ± 7.01	63.2 ± 5.97	0.104
Height (cm)	Mean ± SD	166.57 ± 5.66	166.05 ± 5.23	164.50 ± 3.59	0.384
Sex ratio	Male	16	17	16	1.000
	Female	5	4	4	
Duration of surgery (minutes)	Mean ± SD	122.86 ± 44.90	120.71 ± 19.83	105 ± 17.19	0.153

Sensory and motor block characteristics in the three groups are shown in Table 2. All three groups were significantly different from each other.

**Table 2:** Comparison of sensory and motor block parameters in the three groups. ^€^Denotes that group C is significantly different from both groups N and Cl with a p-value of <0.001. *p-value <0.001 is to denote that all three groups are significantly different from each other.

Parameters		Group N (nalbuphine)	Group Cl (clonidine)	Group C (control)	p-value	Inter-group comparison
Time to T10 (minutes)	Mean ± SD	4.67 ± 0.96	4.76 ± 0.99	6.50 ± 0.88	<0.001^€^	^€^Group C is significantly different from group N and group Cl
Time to achieve maximum level (minutes)	Mean ± SD	8.76 ± 1.17	7.90 ± 1.72	10.8 ± 1.50	<0.001^€^	^€^Group C is significantly different from group N and group Cl
Time to two-seg. regression (minutes)	Mean ± SD	90.71 ± 12.07	141.43 ± 13.88	65.2 ± 8.80	<0.001*	*All three groups are significantly different from each other
Time to reach MBG3 (minutes)	Mean ± SD	8.29 ± 0.71	6.95 ± 1.02	10.10 ± 1.21	<0.001*	*All three groups are significantly different from each other
Time to reach MBG0 (minutes)	Mean ± SD	195.75 ± 17.86	238.57 ± 24.14	165.00 ± 14.60	<0.001*	*All three groups are significantly different from each other

The duration of spinal analgesia and total number of rescue analgesics required in 24 hours are given in Table 3. All three groups were significantly different from each other.

**Table 3:** Comparison of the duration of spinal analgesia and the total number of rescue analgesic doses required in 24 hours. *p-value <0.001 is to denote that all three groups are significantly different from each other.

Parameters		Group N (nalbuphine)	Group Cl (clonidine)	Group C (control)	p-value	Inter-group comparison
Duration of spinal analgesia (minutes)	Mean ± SD	216.75 ± 25.96	292.86 ± 24.94	178.50 ± 16.06	<0.001*	*All three groups are significantly different from each other
Total number of rescue analgesic doses required	Mean ± SD	3.35 ± 0.48	2.67 ± 0.48	4.20 ± 0.52	<0.001*	*All three groups are significantly different from each other

**Figure 2:**
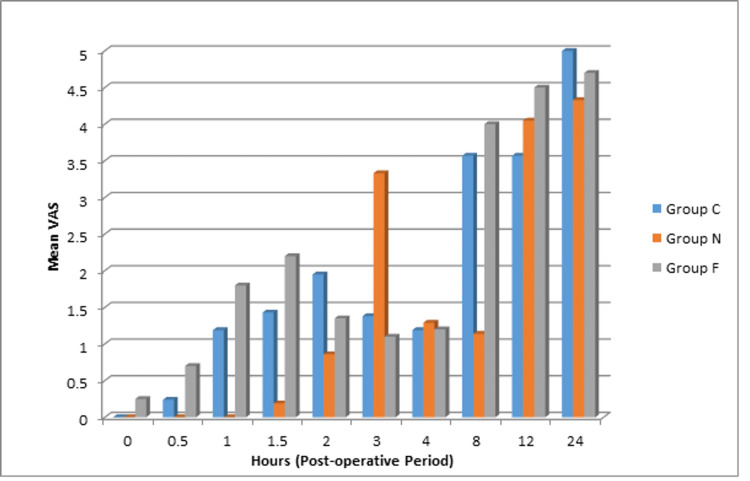
Mean VAS in the postoperative period. VAS: visual analog scale.

The VAS score at different time intervals is shown in Figure 2. It was significantly lower in the clonidine group than the other two groups between one and eight hours in the postoperative period. 

**Figure 3:**
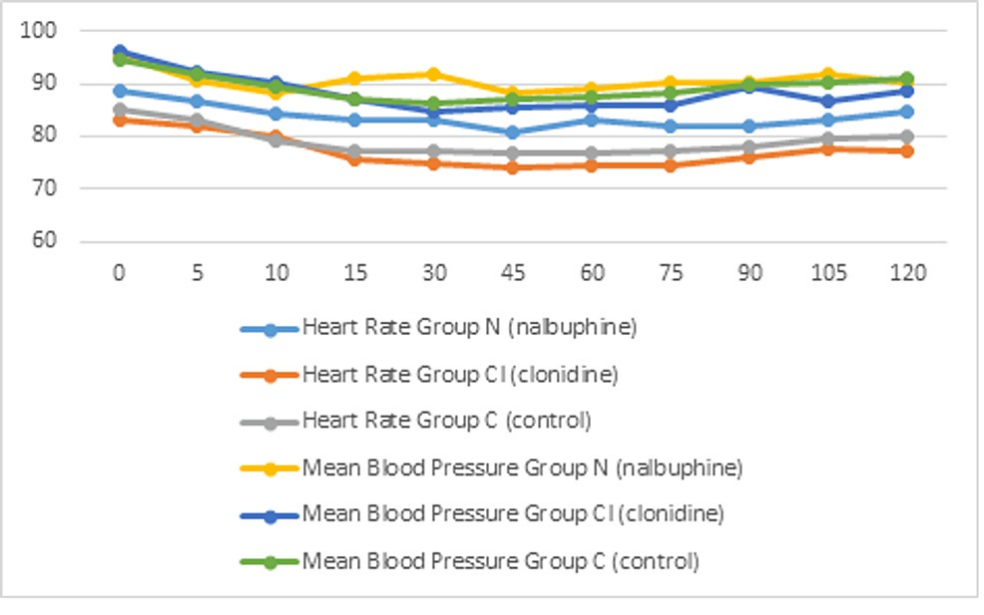
Comparison of intra-operative mean heart rate and blood pressure among the three groups at different time intervals.

Intraoperative heart rate and mean blood pressure at different time intervals are shown in Figure 3. There was a significant difference in heart rate between the nalbuphine and clonidine groups 15 minutes after drug administration. At 30 minutes, there was a significant difference in mean blood pressure in the nalbuphine group with respect to the other two groups. At other intervals, mean blood pressure was comparable among the three groups.

**Figure 4:**
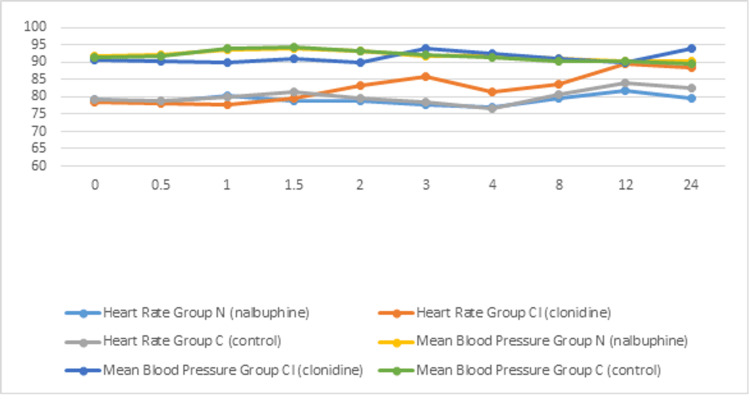
Comparison of postoperative mean heart rate and blood pressure among the three groups at different time intervals.

A comparison of the three groups for adverse effects did not reveal a statistically significant difference. At three, 12, and 24 hours postoperatively, there was a significant difference in mean heart rate between the nalbuphine and clonidine groups. Also, there was a significant difference in mean blood pressure between nalbuphine and clonidine at 24 hours in the postoperative period. A comparison of mean heart rate and blood pressure is shown in Figure 4.

## Discussion

Central neuraxial blockade is the preferred technique of anesthesia for orthopedic lower limb surgeries, but it is often limited due to the short duration of action of local anesthetics. Various opioid and non-opioid drugs have been used to prolong the duration of subarachnoid block [3-9].

Culebras et al. compared 0.2 mg of morphine with 0.2 mg, 0.8 mg, and 1.6 mg of nalbuphine as an adjuvant to 10 mg of hyperbaric bupivacaine 0.5% and concluded that nalbuphine in the dose of 0.8 mg to 1.6 mg when given intrathecally was effective in providing intra-operative analgesia [11]. Sapate et al. also reported a longer duration of analgesia with nalbuphine [20]. Nalbuphine causes activation of supraspinal and spinal κ receptors providing analgesia but can cause minimal respiratory depression and sedation due to the ceiling effect [4,10-15].

Thakur et al. compared different doses of clonidine with normal saline as an adjuvant to hyperbaric bupivacaine for patients undergoing inguinal herniorrhaphy and demonstrated faster onset of sensory and motor block, enhanced quality and duration of analgesia with clonidine [17]. This is due to clonidine acting on α1 and α2 adrenoreceptors as an agonist with predominant α2 action (α2:1-200:1). It produces analgesia at the spinal level by the activation of descending medullospinal noradrenergic tract and central sympatholysis at the presynaptic ganglion, while supraspinally, transduction at the locus coeruleus produces analgesia [4,16,17].

In our study, the onset of sensory block was significantly faster with nalbuphine and clonidine, but the duration was comparable between the two. Similar results were observed by Chetty et al., who used 1.6 mg of nalbuphine and 30 mcg of clonidine [16]. In contrast to our results, Bansal et al. used 2 mg nalbuphine and 30 mcg clonidine and reported faster onset with nalbuphine [4], which might be due to the higher dose of nalbuphine. 

We found significantly prolonged time to two-segment regression and duration of motor block with clonidine as compared with nalbuphine. Similar results were produced by Bansal et al. and Chetty et al. using 2 mg and 1.6 mg nalbuphine, respectively, with 30 mcg clonidine [4,16]. In contrast to our results, Kumar et al. found 0.8 mg of nalbuphine to be superior than 30 mcg of clonidine in prolonging the duration of analgesia and sensory block [19]. We infer that 30 mcg of clonidine provides better sensory and motor block than significantly higher doses of nalbuphine.

In our study, the time taken for the onset of motor block was significantly faster with clonidine as compared to nalbuphine. The mean difference in the onset of motor blockade is only 1.34 minutes between nalbuphine and clonidine, which is not much of clinical importance. Chetty et al. used 1.6 mg of nalbuphine, which may be the reason of reporting comparable onset of motor blockade between nalbuphine and 30 mcg of clonidine [16]. Kumar et al. used 0.8 mg of nalbuphine and 30 mcg of clonidine and observed no significant difference in the onset of motor block with nalbuphine and clonidine [19]. 

In our study, the duration of spinal analgesia was prolonged with clonidine (292.86 ± 24.92 minutes) as compared to nalbuphine (216.75 ± 25.96 minutes). Bansal et al. reported similar results using 2 mg nalbuphine (231.50 ± 26.18 minutes) and 30 mcg clonidine (283.00 ± 14.18 minutes), while Chetty et al. reported the same using 1.6 mg nalbuphine (218.3 ± 35.1 minutes) and 30 mcg clonidine (330.7 ± 47.7 minutes) [4,16].

The difference in duration of analgesia with nalbuphine observed by Chetty et al., Bansal et al., and our study might be due to the use of different doses and study populations. Although different doses of nalbuphine were used in these studies, clonidine was found to be superior in prolonging the duration of analgesia. In contrast to this, Kumar et al. reported prolonged analgesia with only 0.8 mg nalbuphine (273.12 ± 19.80 minutes) when compared with 30 mcg clonidine (175.98 ± 14.66 minutes) [19].

In the present study, the mean VAS score until two hours post-operatively was significantly lower with clonidine than with nalbuphine and control. It was also lower with nalbuphine as compared to the control for 1.5 hours in the postoperative period. The total 24-hour rescue analgesic requirement was less with clonidine than with nalbuphine, whereas it was the maximum in the control group. Similar results were observed by Chetty et al., while Kumar et al. reported that the total number of rescue analgesics required was higher with clonidine [16,19]. We infer from our results that the addition of clonidine and nalbuphine intrathecally to hyperbaric bupivacaine significantly reduces the 24-hour analgesic requirement.

Opioids, when used as an adjuvant in the sub-arachnoid block, are associated with various side effects like hypotension, bradycardia, nausea, vomiting, pruritus, respiratory depression, etc. In our study, the three groups were comparable with respect to the occurrence of side effects. The mean heart rate in the clonidine group was significantly lower than the other two groups at all time intervals, but none of the patients required any therapeutic intervention. At 30 minutes intraoperatively, there was a significant fall in blood pressure in the clonidine group, which was treated with I.V. fluids and injection of mephentermine 6 mg. Clonidine is commonly associated with hypotension due to its effect on α2 receptors and central sympatholysis at the pre-synaptic ganglion. Similar observations were made by Sethi et al., who reported a significant fall in heart rate and blood pressure in patients receiving clonidine as an adjuvant [21]. During the rest of the intraoperative period, the trends of blood pressure monitoring among the three groups were comparable.

Nalbuphine acts on κ opioid receptors as an agonist and as an antagonist at µ receptors and is associated with various side effects like hypotension, nausea, vomiting, bradycardia, sedation, and minimal respiratory depression. Pruritus, a common side effect, is usually seen with intrathecal use of hydrophilic opioids and less commonly with lipophilic opioids. Nalbuphine is a lipophilic opioid and has an antipruritic property. Jyothi et al. also reported no significant increase in the incidence of side effects with nalbuphine. In contrast, Mukherjee et al. reported a higher incidence of side effects with 0.8 mg of nalbuphine as compared to patients receiving lower doses [15,22]. More studies with different doses and larger sample sizes are required to study the side effect profile of nalbuphine when used as an intrathecal adjuvant.

The limitation of the present study would be a specific population with a small sample size. Further studies with a larger sample size and a different study population (those undergoing lower abdominal procedures and inguinal surgeries) need to be conducted.

## Conclusions

In our study, the quality of sensory and motor blocks was better in groups receiving both nalbuphine and clonidine as adjuvants than in the control group. On comparison between clonidine and nalbuphine, patients receiving clonidine had a significantly longer duration of analgesia and a decreased requirement of postoperative analgesia than those receiving nalbuphine. Clinically, the incidence of hypotension was higher in the clonidine group, but it was not significant. All three groups were comparable to each other in terms of side effects. We conclude that clonidine is superior to nalbuphine as an adjuvant to hyperbaric bupivacaine in orthopedic lower limb surgeries without any significant difference in side effects. 
